# Self-Paced Online vs. Cue-Based Offline Brain–Computer Interfaces for Inducing Neural Plasticity

**DOI:** 10.3390/brainsci9060127

**Published:** 2019-06-01

**Authors:** Mads Jochumsen, Muhammad Samran Navid, Rasmus Wiberg Nedergaard, Nada Signal, Usman Rashid, Ali Hassan, Heidi Haavik, Denise Taylor, Imran Khan Niazi

**Affiliations:** 1 SMI, Department of Health Science and Technology, Aalborg University, Aalborg 9220, Denmark; mj@hst.aau.dk; 2 New Zealand College of Chiropractic, Auckland 1060, New Zealand; samran.navid@nzchiro.co.nz (M.S.N.); rasmus.Nedergaard@nzchiro.co.nz (R.W.N.); heidi.haavik@nzchiro.co.nz (H.H.); 3 Mech-Sense, Department of Gastroenterology and Hepatology, Aalborg University Hospital, Aalborg 9000, Denmark; 4 Health and Rehabilitation Research Institute, AUT University, Auckland 0627, New Zealand; nada.signal@aut.ac.nz (N.S.); usman.rashid@aut.ac.nz (U.R.); denise.taylor@aut.ac.nz (D.T.); 5 CEME, National University of Sciences and Technology (NUST), Islamabad 4400, Pakistan; alihassan@ce.ceme.edu.pk

**Keywords:** movement-related cortical potentials, EEG, brain–computer interface, neural plasticity, cortical excitability

## Abstract

Brain–computer interfaces (BCIs), operated in a cue-based (offline) or self-paced (online) mode, can be used for inducing cortical plasticity for stroke rehabilitation by the pairing of movement-related brain activity with peripheral electrical stimulation. The aim of this study was to compare the difference in cortical plasticity induced by the two BCI modes. Fifteen healthy participants participated in two experimental sessions: cue-based BCI and self-paced BCI. In both sessions, imagined dorsiflexions were extracted from continuous electroencephalogram (EEG) and paired 50 times with the electrical stimulation of the common peroneal nerve. Before, immediately after, and 30 min after each intervention, the cortical excitability was measured through the motor-evoked potentials (MEPs) of tibialis anterior elicited through transcranial magnetic stimulation. Linear mixed regression models showed that the MEP amplitudes increased significantly (*p* < 0.05) from pre- to post- and 30-min post-intervention in terms of both the absolute and relative units, regardless of the intervention type. Compared to pre-interventions, the absolute MEP size increased by 79% in post- and 68% in 30-min post-intervention in the self-paced mode (with a true positive rate of ~75%), and by 37% in post- and 55% in 30-min post-intervention in the cue-based mode. The two modes were significantly different (*p* = 0.03) at post-intervention (relative units) but were similar at both post timepoints (absolute units). These findings suggest that immediate changes in cortical excitability may have implications for stroke rehabilitation, where it could be used as a priming protocol in conjunction with another intervention; however, the findings need to be validated in studies involving stroke patients.

## 1. Introduction

A brain–computer interface (BCI) is a device that can decode a user’s intention from voluntary produced brain activity [1]. The decoded activity is then used to control an external device; e.g., speller devices, wheel-chairs, and robotic arms as aids for disabled users, and the control of electrical stimulators and exoskeletons for stroke rehabilitation. It has been established over the past years that BCIs can be used for stroke rehabilitation by strengthening the brain–muscle pathways that were weakened by the stroke, and a likely mechanism for this motor recovery is neural plasticity [2,3]. It is likely that BCI-induced neural plasticity happens through Hebbian-associated and long-term potentiation-like mechanisms where motor cortical activity (movement planning and execution) is temporally correlated with relevant somatosensory feedback [4]. The exact timing between motor cortical activity and afferent inflow for inducing neural plasticity is not known, but it has been speculated that the two events must be paired with an accuracy of less than 200–300 ms [3], which was validated by Mrachacz-Kersting et al. [4]. The motor cortical activity can be elicited by either executing or imagining a movement [5,6,7]. Moreover, stroke patients can also attempt to perform movements which will elicit motor cortical activity [8,9]. Motor cortical activity is manifested in the electroencephalogram (EEG) as a movement-related cortical potential (MRCP) or as sensorimotor rhythms, comprising event-related desynchronization and event-related synchronization [10,11]. To fulfil the strict requirement of temporally correlated somatosensory feedback, the intention to move must be detected before the movement is executed [4]. It is possible to predict the movement by extracting MRCPs or event-related desynchronization on a single-trial basis since they reflect pre-movement activity; this is seen as an increase in negativity for the MRCP, and a power decrease in the alpha/mu and beta band. It has been shown in offline simulation studies that these phenomena can be reliably detected with latencies that allow sufficient time to trigger an external device that can provide relevant somatosensory feedback (e.g., through electrical stimulation or an orthotic device) and for the somatosensory feedback to reach the cortical level [6,7]. In online studies, movement intentions have been detected between −500 and +400 ms with respect to the movement onset [12,13], which may not be optimal for inducing plasticity according to the findings in [4], but the findings in [14,15] support the idea that movement intentions can be detected with latencies that allow the induction of plasticity. It has been established that both self-paced and offline cue-based BCI protocols are efficient for inducing neural plasticity in healthy participants and stroke patients by using exoskeletons and the electrical stimulation of relevant nerves and muscles [4,14,15,16,17,18], improving motor function [17,19,20,21]. The next step in this line of research is to optimize the use of this protocol for rehabilitation in clinical practice. Before BCI technology can be implemented into rehabilitation clinical practice, a number of technical, usability and efficacy issues must be addressed (see [22] for a recent review about the design requirements of BCIs from rehabilitation professionals). Some of these issues include the technical expertise and time required to set up systems, the robustness and calibration of the decoding algorithms, as well as how effectively BCI systems fit with current clinical practice philosophies and workflows, how effectively BCI systems promote the patient’s continued engagement in rehabilitation, and whether BCI systems improve outcomes of importance for people with stroke [23,24].

One key factor which may influence both system usability and efficacy is the BCI mode. Currently, BCI systems can be operated using either self-paced (online) or cue-based (online or offline) modes (also referred to as asynchronous and synchronous). In contrast, cue-based systems are only active during pre-determined periods defined by the presentation of a cue to the user. Besides the online implementation, it can also be implemented as an offline/associative BCI [4]. In self-paced systems, the BCI is always active and is required to accurately detect a defined change in brain activity from continuous data. Self-paced systems offer advantages that could be appealing in the rehabilitation context. Firstly, the user has control of the system which may promote engagement with BCI rehabilitation, and secondly, the BCI could be embedded in usual rehabilitation activities or activities of daily living, which may strengthen its fit with clinical practice and extend the reach of BCI rehabilitation beyond defined sessions. However, the disadvantage of self-paced BCI systems is that the performance of the system can be affected by the need to be always active, resulting in a trade-off between having a high true positive rate and a low number of false positive detections per minute. 

Both cue-based and self-paced systems have been shown to induce neural plasticity. The induction of plasticity has been in the range of a 35%–175% increase in corticospinal excitability for the offline cue-based BCI [4,16,17], while increases of 50%–100% have been reported with an online self-paced system [14,15]. However, these results come from disparate research using different feedback modalities, electrical stimulation or exoskeletons and participants, stroke patients and healthy participants. It is unclear whether decrements in system performance inherent in online systems influence the neurophysiological or clinical effects of BCI or whether this disadvantage is outweighed by other advantages. The disadvantage of the current offline cue-based system [17] is that it is calibrated in a training session from which the trigger timing of the external device is fixed with respect to a cue. If the users change their motor strategy during the testing session, the timing between the movement intention and somatosensory feedback may be mismatched; e.g., they will still receive electrical stimulation even though they did not imagine or attempt to perform a movement.

Therefore, the aim of this study was to compare the effect of self-paced and cue-based motor output modes on neural plasticity during a MRCP-driven BCI.

## 2. Methods

### 2.1. Participants

Seven women and eight men participated in the study (25 ± 4 years old). The participants gave written informed consent, which conformed to the Declaration of Helsinki, and the study was approved by the Northern B Regional Ethics Committee of New Zealand (14/NTB/113). All participants were screened for contraindications to transcranial magnetic stimulation (TMS) [25].

### 2.2. Measurements and Stimulation

#### 2.2.1. EEG

Continuous EEG was recorded using a 64-channel amplifier (TMSi) with a sampling frequency of 2048 Hz. The channels F3, Fz, F4, C3, Cz, C4, P3, Pz, and P4 were used for the BCI operation. Electrooculography was recorded from FP1. All channels were referenced to the left mastoid (M1). The impedance of the electrodes was below 10 kΩ. The participants were instructed to minimize activity in facial muscles and eye movements. 

#### 2.2.2. Motor-Evoked Potentials

**Figure 1:**
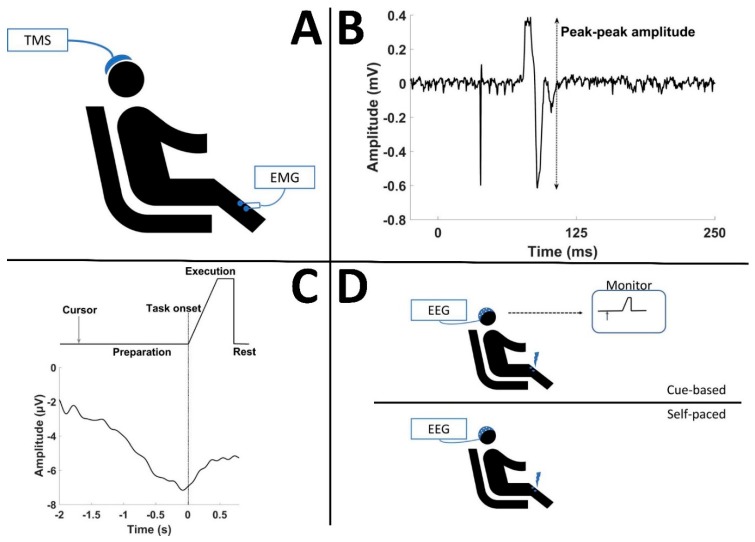
(**A**) Experimental setup for transcranial magnetic stimulation(TMS) measurements. (**B**) Extraction of the peak–peak amplitude of a motor-evoked potential (MEP). (**C**) Visual cue that was presented to the participants and an example of an averaged movement-related cortical potential (MRCP) from the 50 movements prior to the cue-based brain–computer interface (BCI) training. Note that in this example the peak negativity occurs prior to the task onset, and it is this latency of peak negativity with respect to the task onset that is considered to be stable throughout the cue-based BCI training. (**D**) The participants receive electrical stimulation when they imagine a movement. There is no visual cue provided in the self-paced BCI training.

Motor-evoked potentials were elicited in the right tibialis anterior (TA) using a transcranial magnetic stimulator (Magstim 200, Magstim Company, Dyfed, UK) and a figure-of-eight double cone coil with a posterior–anterior current direction. Single-pulse TMS was applied. MEPs were recorded using electromyography (EMG) with (Ag/AgCl Neuroline 720, Ambu, Denmark) electrodes placed on the belly of the muscle. The EMG was sampled at 4000 Hz, amplified, and band-pass filtered from 20–1000 Hz (using a custom-made EMG amplifier). The peak–peak amplitude of the MEP was extracted and used in the analysis (see Figure 1A,B). 

#### 2.2.3. Transcranial Magnetic Stimulation

The TA hotspot was identified by altering the coil position until the site eliciting the largest MEP was identified. This location was marked and recorded so the coil could be placed in the same location throughout the experiment. The resting motor threshold (RMT) was then determined. The RMT was defined as the lowest stimulator output where 5 out of 10 MEPs exceeded 50 µV. In the recordings, 15 stimuli were delivered at 120% RMT; each stimulus was separated by 5–7 s. All the trials were visually inspected, and the trials which had EMG activity in the pre stimulus part were rejected.

#### 2.2.4. Peripheral Nerve Stimulation

Single pulse electrical stimulation was applied to the deep branch of the right common peroneal nerve. The surface stimulation electrodes were placed with the cathode proximal and the anode distal. The electrodes were placed in such a way that there was only activity in the TA and not in any synergistic or antagonistic muscles; this was checked by palpation. The nerve was stimulated at the motor threshold, which was defined as the lowest amount of current that would elicit a palpable response in the tibialis anterior tendon. The pulse width was 1 ms, and a Digitimer Stimulator DS7AH was used to deliver the stimulus.

### 2.3. Experimental Setup

The experiment consisted of two experimental BCI training sessions: 1) cue-based and 2) self-paced. The order of the BCI training sessions was randomized, and the two sessions were separated by at least 24 h. At the beginning of the sessions, the participants were asked to imagine 50 dorsiflexions of the right ankle joint while EEG was recorded. The participants were cued when to initiate the movement imagination; this cue was also used in the calibration of the cue-based system. The BCI systems are described below. The initial EEG recordings were followed by a pre-measurement using TMS, which was repeated immediately after the BCI training session and 30 min later. In the BCI training sessions, 50 peripheral electrical stimuli were paired with the movement intention.

### 2.4. Brain–Computer Interface Systems

The data were acquired from the amplifier and processed using code developed in MATLAB 2015b (The MathWorks, Inc., Natick, MA, USA.). The continuous EEG recordings were band-pass filtered between 0.5 and 10 Hz with a zero-phase 2nd-order Butterworth filter. The filtered data was down-sampled to 32 Hz to reduce the computation time of the rest of the steps. A surrogate channel was obtained by using an optimized spatial filter [6]. Afterwards, the data were epoched with respect to the cue (cue-based BCI) or EMG onset (self-paced BCI). In the online self-paced BCI training, the abovementioned pre-processing was applied on 2-s data segments. New data were imported every 200 ms. The complete procedures are summarized below, and greater detail can be found in [4] for the cue-based system and in [14] for the self-paced system.

#### 2.4.1. Cue-Based BCI (Offline)

The processed EEG was divided into epochs based on the visual cues that were provided to the participants (see Figure 1C). The epochs were averaged, and the peak of maximum negativity was identified. The latency between the peak of maximum negativity and the visual cue to initiate the imagined movement was calculated. In the BCI training session, the electrical stimulation was delivered with the following timing: “visual cue—latency—50 ms” [4]. Fifty milliseconds are subtracted due to the conduction time of the afferent stimulus and central processing delays in the brain [4,26].

#### 2.4.2. Self-Paced BCI (Online)

The EEG from the 50 movements that were recorded during the calibration phase was used to individualize the self-paced BCI system as described in Niazi et al. [14]. The subjects were asked to leave a gap of few seconds between the movements. The EEG was pre-processed as described above, and movement intentions were detected using a matched-filter approach [6]. The calibration data were used to build a detection template of the initial negative phase of the MRCP and to determine the threshold for movement detection. The threshold was determined from a receiver operating characteristics curve to obtain a trade-off between the number of false positive detections and the true positive rate (TPR). The system was disabled for 5 s after the system detected a movement intention to ensure at least a 5-s rest period between pairings. Moreover, the detector was disabled if the eye (EOG) activity in FP1 exceeded 125 µV.

During the period in which the detector was disabled, the participants extended their right index finger for ~2 s after their imagined movement if it was a true positive detection. They extended their left index finger if it was a false negative. Movement of the index fingers was used to avoid/reduce movement artefact from the jaw muscles during the speech.

The performance of the system was evaluated using the TPR, number of false positive detections per minute (FPs/min), and the number of false negatives per minute (FNs/min). These were not calculated for the cue-based system since it relied on the participants being able to consistently time the motor imagination to the visual cue [4].

### 2.5. Statistics

The following were investigated: 1) whether the pre- to post- and 30-min post-treatment effects of the self-paced and cue-based BCI systems induced cortical excitability changes, and 2) the difference in these effects across the two systems.

For all of the MEP analyses, the peak–peak amplitude was extracted from each MEP and averaged across the 15 repetitions. The analyses were performed in absolute units (peak–peak amplitude: mV) and in relative units (%). The relative change in MEP amplitudes was calculated as (post − pre)/pre- × 100.

Linear mixed regression models were used for the analyses, and they were performed in R version 3.5.1 (R Foundation for Statistical Computing, Vienna, Austria). The advantages of the linear mixed regression models over the traditional analysis of variance (ANOVA) approach have been studied in multiple related research areas [27,28,29]. In this case, the linear mixed regression approach had advantages over ANOVA as it allowed us to model the data with a Gamma distribution to evaluate differences in millivolts instead of evaluating differences in log-millivolts because the data was not normally distributed, adjust for baseline differences to estimate the pre- to post-effect sizes, and account for correlated repeated measures. In addition to these benefits, the linear mixed models used here may allow the evaluation and inclusion of other covariates in a larger randomised controlled trial based on this study. The evaluation and inclusion of covariates was done in a similar study which was published earlier using the same brain–computer interface [30]. The *lme4* package version 1.1-21 was used to fit all the models [31]. The model for the absolute units was

(1) $$\begin{array}{c} MEP_{abs}\sim 1+MEP_{pre}+Session+Time+Session:Time\\ +(1│Subject:Session) \end{array}$$

MEP amplitudes were estimated across the two sessions (self-paced, cue-based) at the time points of post-and 30-min post-intervention with respect to the pre-MEP amplitudes. A similar model has been suggested previously [32], but two changes have been made: 1) the subjects across the sessions were the same and we assumed that they responded differently to the two interventions—therefore, the subject- and session-wise random intercept (1│Subject:Session) were used to estimate the between-subject variance in each session separately (this also fits the repeated measures study design); and 2) Gamma distribution was used to model the data as well as the log-link function. The selection of the model structure was based on Akaike information criterion corrected for small samples. This was done to avoid under-fitting and over-fitting. Other candidate model structures which were considered were ones with a Gamma distribution and identity link and with only a subject-wise random intercept (*1|Subject*).

The same model was used as in Equation 1 for relative units, but the Gaussian distribution and identity-link modelled the data as percentage changes instead. The model is given below.

(2) $$\begin{array}{c} MEP_{\%}\sim 1+MEP_{pre}+Session+Time+Session:Time\\ +(1|Subject:Session) \end{array}$$

Significance was assumed when *p* < 0.05. Using the *emmeans* package version 1.3.3 [33], pre- to post-effect sizes with standard errors were obtained from models 1 and 2 as marginal means at MEP_pre_ = 0, and their pair-wise contrasts were investigated using Tukey’s HSD(honestly significant difference) method [34].

## 3. Results

### 3.1. MEP Size

**Figure 2:**
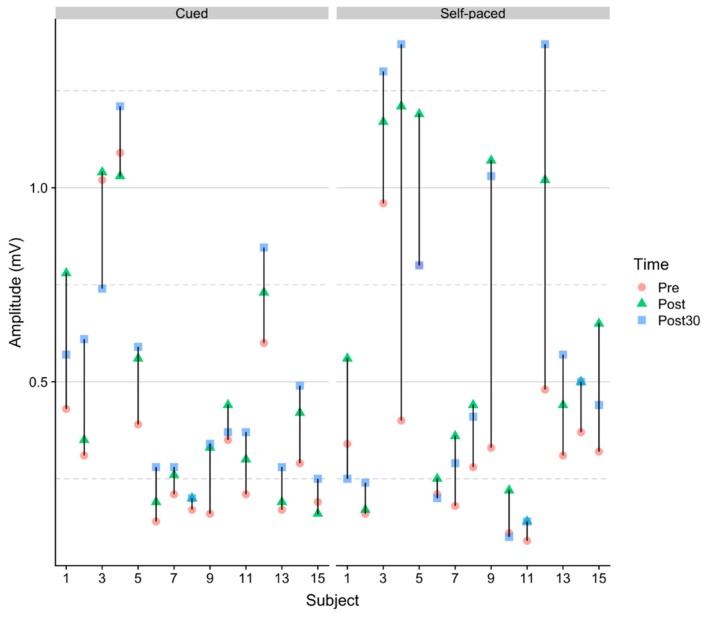
Peak–peak raw MEP amplitudes for the subjects.

**Figure 3:**
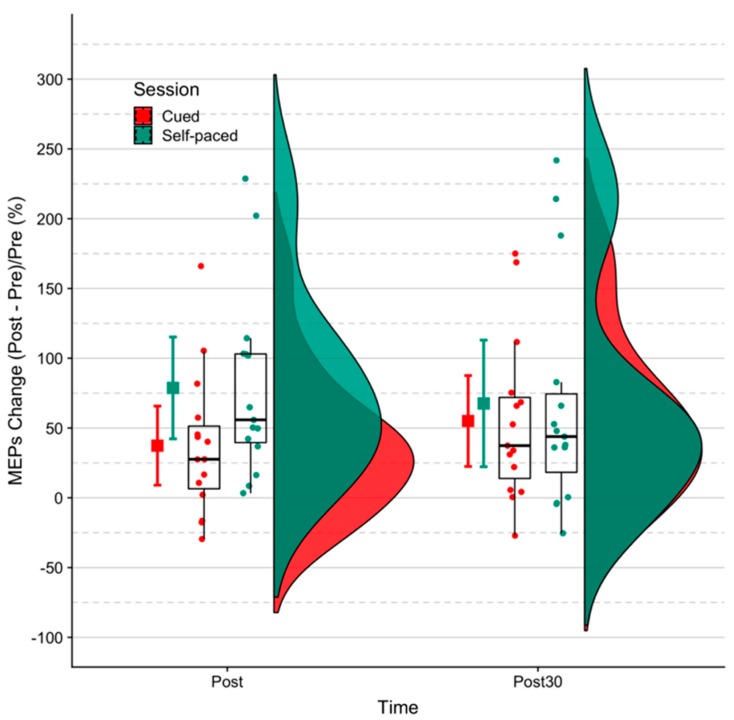
Percentage changes in MEP peak–peak amplitudes calculated for each subject. Error bars show mean ± 95% confidence interval (CI).

**Figure 4:**
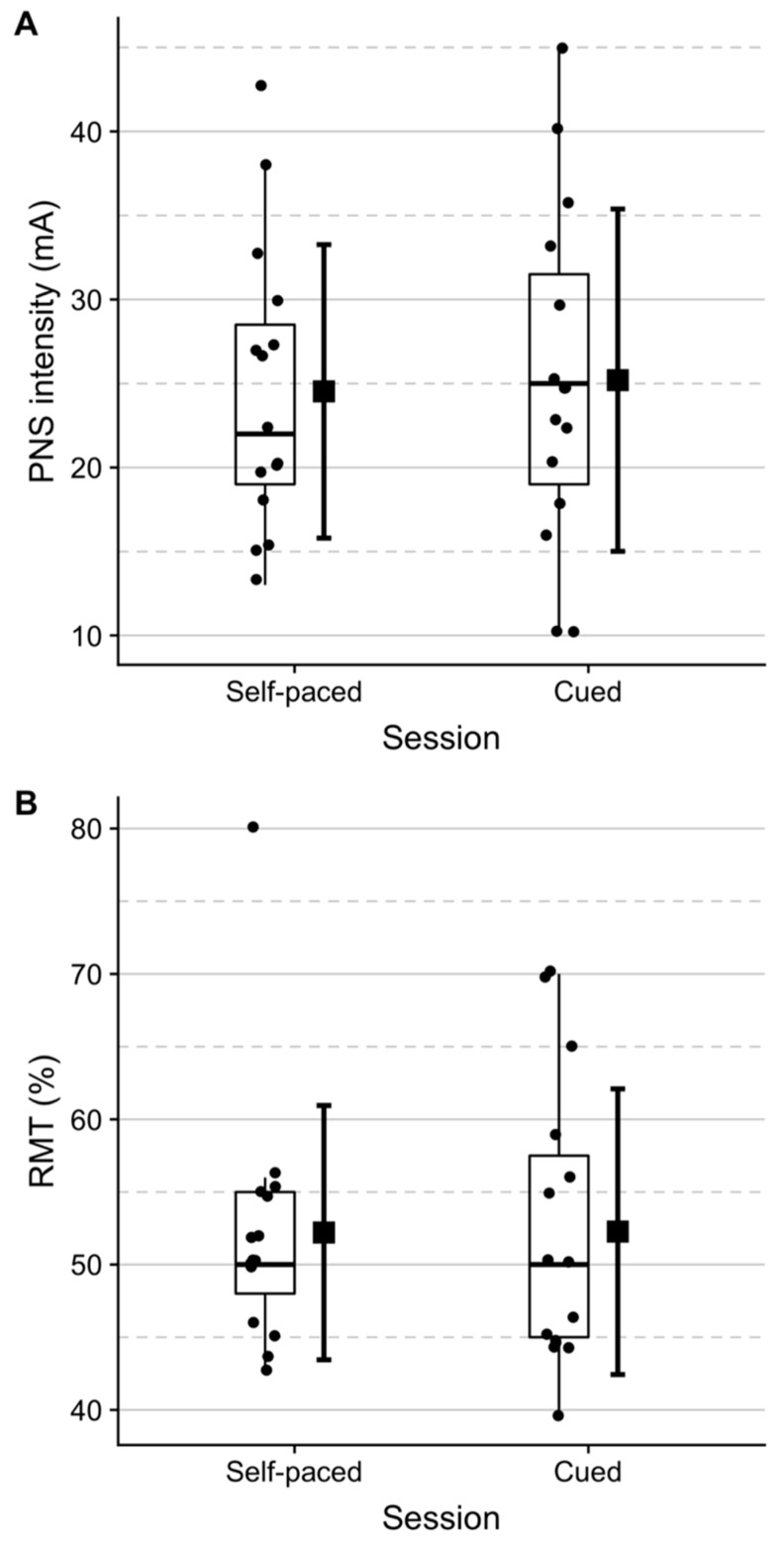
(**A**) Peripheral nerve stimulation (PNS) intensity in mA and (**B**) TMS machine resting motor threshold (RMT) output in % for each subject. Error bars show mean ± SD.

The MEP amplitudes for all subjects are plotted in Figure 2 and relative changes in raw MEP amplitudes are shown in Figure 3. The trends of each participant suggest that the MEP amplitude increases more from pre- to post-intervention for the self-paced BCI training. Mean intensity values for RMT and peripheral nerve stimulation across subjects are given in Figure 4. 

The effect sizes based on the statistical models from pre- to post-intervention are presented in Table 1, and the pair-wise contrasts across the sessions are given in Table 2. The MEP amplitudes in absolute units were modelled using log-link; thus, the contrasts on the log scale are shown on a response scale. 

These results revealed that both BCI systems increased (*p* < 0.05) the MEP amplitudes significantly from pre- to post- and 30-min post-intervention when using absolute units and relative units. However, the effect sizes of the self-paced system were larger than the cue-based system. In relative units, MEPs were significantly different (*p* = 0.03) between the two systems post-intervention but not at 30-min post-intervention. 

### 3.2. BCI Performance

In the cue-based intervention, the average time across all subjects for movements relative to the cues was 11.73 ± 165 ms. The performance of the self-paced BCI is summarized in Table 3. On average, 75% of the movement imaginations were correctly detected at ~1 FP/minute. To reach 50 correct stimuli, an average of 67 movements were required. The inter-movement intervals in the training phase of online BCI was 11.48 ± 1.11 s.

## 4. Discussion

The results of this study indicate that a self-paced MRCP-driven BCI system led to increased corticospinal excitability immediately following the intervention when compared to a cue-based BCI system. However, these system differences were not sustained at 30 min. The findings suggest that both self-paced and cue-based BCI systems are effective at promoting neural plasticity.

The BCI performance of the self-paced system was similar to those reported previously [13,14], demonstrating that MRCPs can be detected without any prior training in naïve BCI users [35]; however, the performance of the system could be optimized so that it would be possible to obtain more correct pairings in a shorter duration of time, making the training more effective. An improvement of the system performance can potentially be obtained by using more robust decoding algorithms or by using movement execution instead of motor imagination [8], which would be the scenario in the clinic, where patients will be asked to attempt to execute the movement instead of imagining it. It is also possible to obtain greater motor cortical activation when a movement is executed instead of being imagined, which is reflected in a larger amplitude of the MRCP when comparing motor execution with motor imagination [7]. Moreover, an online cue-based BCI could be considered, which would solve the potential issue of incongruent pairings between motor cortical activity and afferent feedback from the electrical stimulation; this would also reduce the number of false positives. These speculations must be tested in studies involving stroke patients and in real world scenarios outside the controlled settings in the laboratory. For the cue-based system, it was possible for naïve users to operate this as well without any prior training. The advantage of the cue-based approach in this study is that the amount of technical optimization of the system is less than that of the self-paced system, which is likely to be more appealing in a clinical setting [23]. Moreover, the cue-based intervention was slightly shorter since only 50 stimuli were delivered, corresponding to a TPR of 100 % and 0 false positive detections per minute.

This research indicates that the trade-offs associated with increased system error in self-paced BCI systems are outweighed by enhanced neurophysiological benefits when the user determines the pace of motor imagination. However, it is not clear what drives this difference. When using cue-based systems it has been noted that there is a standard deviation of 200–300 ms of the onset of the imagined movement with respect to the cue within a single session [4,18], so the users cannot always time the imagined movement perfectly with respect to the cue. This could especially be problematic in-patient populations where cognitive and perceptual deficits may impair the ability to attend to the task and predict the timing of the cue. In cue-based systems, failure to attend to the cue accurately can lead to dissociation of the temporal correlation between the afferent stimulus and the motor cortical activity. This issue is potentially suppressed by self-paced systems where the user is not required to respond to an external cue and controls the pace. It is likely that the number of accurate pairings of afferent stimulation and motor cortical activity is higher in the self-paced mode; this may account for the differences seen in the current study. It would be interesting to investigate this hypothesis, but identifying the peak of maximal negativity precisely is difficult on a single-trial level due to the low signal-to-noise ratio.

This study validates previous findings that cue-based and self-paced EEG-triggered electrical stimulation can increase the motor cortical excitability [4,14,15,17,18], and in a very recent study, the two systems were compared with similar results [36]. It was not tested if imagination alone and stimulation alone would change the motor cortical excitability, but it has been shown previously that a similar number of imaginary movements and stimuli do not change the motor cortical excitability [4,14]. It is important to note that there were substantial inter-individual differences in effect, which can be seen in Figure 2. Several factors can contribute to this inter-subject variability, such as aerobic exercise level, attention and time of the day for details (see [37] for a review). The high inter-subject variability is also reflected in the stimulation thresholds which are presented in Figure 4. The variability in Figure 4 is also attributed to anatomical differences since the depth of the common peroneal nerve and the representation of foot may differ amongst the participants. The larger increase for the self-paced intervention could be that the subject had to pay attention to trigger the stimulation contrary to the cue-based intervention where the subject would receive electrical stimulation independent of the brain state; however, this is a speculation, and there was no difference between the interventions after 30 min. It was only in the cue-based BCI training that visual feedback was provided. It was not investigated whether the participants felt it was easier to perform motor imagination when visual feedback was presented. The results do not suggest a difference, at least in terms of the induction of plasticity. However, it is likely that some stroke patients may benefit from a visual cuem while others, potentially those with cognitive impairments, will be distracted by the visual feedback. This hypothesis has not been tested. The factors outlined in [37] are expected to explain some of the differences that are observed between the changes in excitability in this study compared to previous studies [4,14,15,18] when using TMS to evoke MEPs, which also leads to large standard errors. Another factor that could affect the induction of plasticity is the ability to imagine movements and in response trigger electrical stimulation, which is a skill that can potentially be improved with training [1]. All the subjects responded positively to the self-paced intervention from pre- to post-intervention measurement, while three subjects had a decrease from pre- to post-intervention measurement in the cue-based intervention (see Figure 2). However, these three subjects had an increased MEP amplitude in the 30-min post-intervention measurement compared to the pre-intervention measurement, which could reflect that it takes some time for the changes in cortical excitability to consolidate. In the previous work, this protocol has been shown to generate a high percentage of subjects who had an increase in cortical excitability, but it should be noted that only 8 subjects were included [14]. Based on the results in this study, there is an indication of a high level of responders compared to, for example, traditional paired associative stimulation, where higher levels of non-responders have been reported [38]. However, it is difficult to compare the efficacy of the many different neuromodulation protocols due to their methodological differences in terms of stimulation sites and parameters [37,39,40]. It seems that both self-paced and offline cue-based BCI protocols are efficient in inducing plasticity with a relatively low number of repetitions, and the contraindications associated with TMS are avoided [25].

## 5. Conclusions

Both cue-based and self-paced BCIs can be used to induce neural plasticity. The self-paced approach led to higher immediate changes in the neural plasticity compared to the cue-based approach; however, there was no statistical difference 30 min after the intervention. The findings in the current study should be validated in a stroke population to determine if the same neuroplastic changes are observed, and if the patients can initiate movements with precise and consistent timing according to the visual cues.

## Figures and Tables

**Table 1:** The effect sizes and standard errors are presented based on the statistical models. H_0_ = 0 stands for the null hypothesis; i.e., that the effect size is 0.

**Session**	**Time**	$MEP_{abs}\;(\mathrm{mV})$	**Standard Error (mV)**	$z,\;p,\;H_{0}=0$
Self-paced	post-	0.25	0.05	*z* = −7.44, *p* < 0.001
Cue-based	post-	0.18	0.04	*z* = −8.53, *p* < 0.001
Self-paced	post 30-	0.22	0.04	*z* = −8.05, *p* < 0.001
Cue-based	post 30-	0.21	0.04	*z* = −7.95, *p* < 0.001
**Session**	**Time**	$MEP_{\%}$ **(%)**	**Standard Error (%)**	$t\left[df\right],\;p,\;H_{0}=0$
Self-paced	post-	93.26	19.82	*t*[31.91] = 4.71, *p* < 0.001
Cue-based	post-	44.66	20.46	*t*[31.58] = 2.18, *p* = 0.04
Self-paced	post30-	80.72	19.82	*t*[31.91] = 4.07, *p* < 0.001
Cue-based	post30-	62.51	20.46	*t*[31.58] = 3.05, *p* < 0.01

**Table 2:** The contrasts and standard errors are presented based on the statistical models. H_0_ = 1 and H_0_ = 0 stand for the null hypotheses; i.e., that contrasts of the effect sizes are 1 and 0, respectively. On the ratio scale, 1 implies that the two effect sizes are equal, whereas on the linear scale, 0 implies equal effect sizes.

**Time**	**Self-Paced/Cue-Based (Ratio)**	**Standard Error (Ratio)**	$z,\;p,\;H_{0}=1$
Post-intervention	1.34	0.27	*z* = 1.46, *p* = 0.15
30-min post-intervention	1.06	0.21	*z* = 0.29, *p* = 0.77
**Time**	**Self-Paced − Cue-Based (%)**	**Standard Error (%)**	$t\left[df\right],\;p,\;H_{0}=0$
Post-intervention	48.59	21.04	*t*[36.2] = 2.31, *p* = 0.03
30-min post-intervention	18.21	21.04	*t*[36.2] = 0.87, *p* = 0.39

**Table 3:** BCI system performance for self-paced motor imagination.

Participant	True Positive Rate (%)	Number of False Positive Detections per Minute	Duration of the BCI Intervention (min)	Total Number of Movements Performed
1	74	0.8	12	68
2	79	0.2	14	63
3	77	1.0	7	65
4	77	0.4	19	65
5	72	2.0	15	69
6	74	0.8	13	68
7	78	1.0	14	64
8	81	2.0	11	62
9	72	0.4	19	69
10	78	1.7	16	64
11	72	1.5	11	69
12	69	1.3	12	72
13	70	0.3	21	71
14	74	1.2	9	68
15	79	2.7	13	63
Mean ± Std	75 ± 3	1.2 ± 0.7	14 ± 4	67 ± 3
